# Transoral Robotic Surgery for Early-T Stage Glottic Cancer Involving the Anterior Commissure—News and Update

**DOI:** 10.3389/fonc.2022.755400

**Published:** 2022-01-31

**Authors:** Chen-Chi Wang, Wen-Jiun Lin, Jing-Jie Wang, Chien-Chih Chen, Kai-Li Liang, Yen-Jung Huang

**Affiliations:** ^1^ School of Medicine, National Yang Ming Chiao Tung University, Taipei, Taiwan; ^2^ Department of Otolaryngology-Head & Neck Surgery, Taichung Veterans General Hospital, Taichung, Taiwan; ^3^ Department of Audiology and Speech-Language Pathology, Asia University, Taichung, Taiwan; ^4^ Institute of Medicine, Chung Shan Medical University, Taichung, Taiwan; ^5^ Department of Radiation Oncology, Taichung Veterans General Hospital, Taichung, Taiwan; ^6^ Ph.D. Program in Translational Medicine, National Chung-Hsing University, Taichung, Taiwan

**Keywords:** cancer, glottis, larynx, radiotherapy, survival, anterior commissure, swallowing, transoral robotic surgery

## Abstract

**Background:**

About 20% of all glottic carcinomas involve the anterior commissure (AC), and AC involvement was deemed to be a risk factor of local recurrence and poor prognosis. Transoral robotic surgery (TORS) has been developed for a panoramic view of the AC and en-bloc resection of the tumor by multidirectional dissection with endo-wristed instruments. With satisfactory preliminary results, we would like to update the data with a bigger cohort and present the news on using TORS for salvage treatment of recurrence from irradiation failure.

**Methods:**

From July 2010 to December 2019, 22 patients with early T1 and 2 stage primary (n = 11) or recurrent (n = 11) glottic cancer with AC involvement received TORS without adjuvant therapy. TORS exposure was found to be better than TLM by conventional laryngoscopy in diagnostic biopsy. Seven of the 22 patients had recurrent cancer from irradiation failure. The perioperative factors that may be associated with survival were retrospectively analyzed, and the 5-year overall survival (OS)/disease-specific survival (DSS)/recurrence-free survival (RFS)/and organ preservation survival (OPS) rate were estimated by the Kaplan–Meier Method. Their voice and swallowing functions were evaluated by questionnaires of Voice Handicap Index-10 (VHI-10) and Functional Outcome Swallowing Scale (FOSS).

**Results:**

All 22 TORSs were completed smoothly. After a mean follow-up of 49 ± 35.9 months, the Kaplan–Meier method estimated 5-year OS/DSS/RFS/OPS was 93.8%, 93.8%, 74.6%, and 86.3%, respectively. Our 11 patients with fresh cancer had 100% recurrence-free survival. Although the recurrent rate was higher in patients with history of RT, they could be rescued by further open laryngectomy without compromising the OS and DSS. Only one patient expired. The other 21 patients had satisfactory swallowing function with FOSS of 0.33 ± 0.66. Five patients depended on tracheostomy, but the rest 17 patients had serviceable voice with VHI-10 of 18.41 ± 11.29.

**Conclusions:**

TORS could be used in the primary or salvage management of glottic cancer with AC involvement while TORS was confirmed to have better exposure to TLM. The RFS was good for patients with primary cancer. In patients having irradiation failure, TORS could also be a minimally invasive transoral approach before trying open surgery to preserve the organ.

## 1 Introduction

The anterior commissure (AC) of glottis is the location at which bilateral vocal folds join anteriorly to the angle between the laminae of the thyroid cartilage (1). It has been reported that approximately 20% of all glottic tumors involve the AC (2). This is because Broyles’ ligament inserts into the thyroid cartilage at AC, and penetration might induce susceptibility to tumor invasion. In addition, there is only a 2–3-mm space between the AC mucosa from the thyroid cartilage. The lack of a thyroidal perichondrium in this area and vascularization due to ossification of the thyroid cartilage further advance the possibility of ventral tumor spread and infiltration of thyroid cartilage at AC. Therefore, AC involvement was deemed to be a risk factor of glottic cancer recurrence (3).

The conventional treatment of early glottic cancer includes open partial laryngectomy (PL), transoral laser microsurgery (TLM), and radiotherapy (RT) (4). Although radiotherapy is a popular organ preservation therapy, Bron et al. (5) reported that surgery provides better initial local control and final laryngeal preservation compared to RT. After radiotherapy failure, many patients can be salvaged by surgery, but a total laryngectomy (TL) may be required (1). Sachse et al. showed that there is no difference in local control between open PL and TLM (6). Therefore, open PL is mostly reserved for recurrence at the present time (7) and TLM decreases the chance of postoperative morbidity.

The role of TLM for early glottic cancer has been popularized by Steiner in the 1980s, and he also reported his experience with tumors involving the AC with excellent results (8). However, Eckel et al. identified that tumors located at or involving the AC had the highest site for local recurrence (37.1%) of all sites in the larynx (9). In one of the pioneered studies on using TLM for 16 patients in salvage surgery after radiotherapy failure in T1 and T2 glottic carcinoma reported by Puxeddu et al. (10), two of three (66.6%) patients with AC involvement developed a second recurrence. They concluded that involvement of the AC in recurrent glottic cancer after radiotherapy seems to be a relative contraindication to TLM. Inadequate exposure and tangential visualization leading to incomplete excision are the main reasons for the higher recurrence rate. As an endoscopic approach, TLM has several limitations, such as a narrow working field, the need for long and cumbersome instruments, and the line-of-sight issue of laser beam. To conquer the limitation, transoral robotic surgery (TORS) for glottic cancer was first demonstrated by O’Malley et al. in a canine model (11). TORS do not have any line-of-sight limitation from wristed instruments; therefore, there is an increased degree of freedom. The da-Vinci surgical robot system also offers the advantage of 3D high magnification with motion scaling and tremor filtration functions for endoscopic surgery. In January 2010, the U.S. Food and Drug Administration approved TORS for use in benign and malignant diseases of the tonsils, pharynx, and larynx.

Several series of patients with early T-stage glottic cancer treated by TORS have been published (12–18), but the issue of AC involvement has seldom been addressed. Our preliminary report in 2016 demonstrated no local recurrence on using TORS for 8 patients of AC involvement (17). However, we are not sure yet about the feasibility of TORS for salvage surgery after irradiation failure. In this study, after using TORS for a larger cohort of 22 patients, we aimed at updating our long-term results and presenting the news for recurrent patients after TLM and/or radiotherapy failure. The advantages and disadvantages of TORS are further discussed.

## 2 Material and Methods

**Figure 1:**
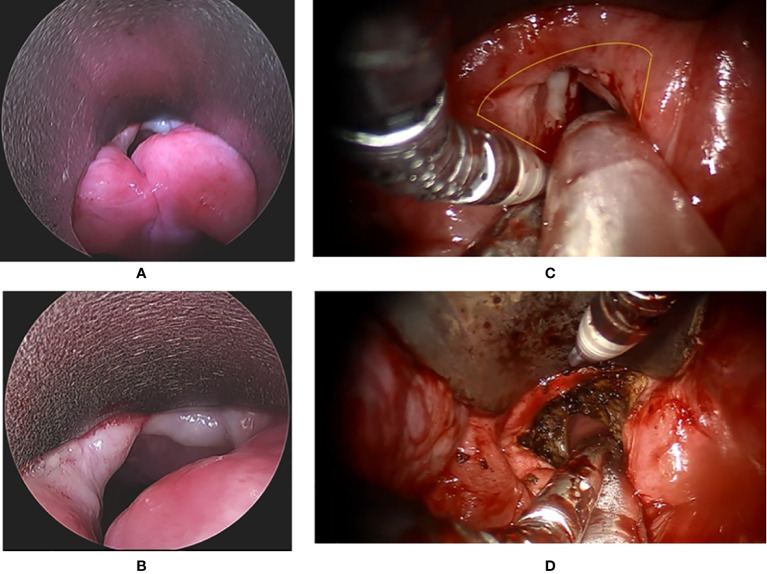
The endoscopic view of a patient with recurrent glottis cancer with AC involvement after radiation failure during conventional TLM biopsy and TORS. **(A)** Zoom-out view of TLM with limited exposure of AC and false vocal folds. **(B)** Zoom in view of TLM with limited exposure. **(C)** Zoom-in view of TORS with panoramic exposure of AC and bilateral false vocal folds. The yellow line indicates the planned resection margin. **(D)** Zoom-out view of TORS after the cancer involving AC and false vocal folds was resected. .

From July 2010 to December 2019, 22 patients with early T-stage primary or recurrent glottic cancer with AC involvement received TORS in our institute. All participants signed written informed consent to receive the surgery, and the Institutional Review Board of Taichung Veterans General Hospital approved this retrospective study (protocol number CE20389B). The perioperative data about the 22 patients are summarized in **Table 1** with 7 T1 and 15 T2 cancers. Eleven patients with recurrent cancers had history of laryngeal cancer after TLM (n = 4) or radiotherapy (n = 5) or both (n = 2) either in our hospital or in other institutes. The rest of the 11 patients had early glottic cancer without treatment before. While confirming their diagnosis of cancer, we tried Feyh–Kastenbauer (F–K) Retractor System (Gyrus Medical, Maple Grove, MN), Laryngeal Advanced Retractor System (LARS; Fentex, Tuttlingen, Germany), or other similar retractor systems that may be used in future TORS during conventional laryngoscopy-guided biopsy under general anesthesia. If the tumor exposure was better with F–K or LARS systems than with the conventional direct laryngoscopy (**Figure 1**), and the mouth could be opened at least 3.5 to 4.0 cm in height (17), then TORS was recommended. All the patients received preoperative images studies including CT and MRI, and there was no clinical evidence of thyroid cartilage invasion in all 22 patients. There was no suspicion of neck metastasis either in 20 patients. If there was evidence of possible cervical metastasis, TORS with neck dissection was performed simultaneously. Otherwise, patients only received TORS.

**Table 1:** The summarized data of overall 22 patients and their outcomes.

	Total (n = 22)	
	n	%
Age (mean ± SD)	66.55 ± 8.96	
Sex		
Female	1	(4.55%)
Male	21	(95.45%)
Clinical T stage		
I	7	(31.82%)
II	15	(68.18%)
Clinical N stage		
0	20	(90.91%)
I	1	(4.55%)
II	1	(4.55%)
Clinical stage		
I	7	(31.82%)
II	13	(59.09%)
III	1	(4.55%)
IV	1	(4.55%)
Cordectomy type		
Va+c	19	(86.36%)
VI	3	(13.64%)
Pathologic stage		
I (T1N0)	9	(40.91%)
II (T2N0)	11	(50.00%)
III (T3N0)	2	(9.09%)
Cancer differentiation		
md	10	(45.45%)
md to pd	8	(36.36%)
pd	4	(18.18%)
Permanent specimen margin		
Negative	13	(59.09)
Positive or undetermined	9	(40.91)
Lymphovascular invasion		
No	20	(90.91%)
Yes	2	(9.09%)
Outcome (5 year)		
Death	1	(4.54%)
Disease-specific death	1	(4.54%)
Recurrence	5	(22.7%)
Total laryngectomy	3	(13.6%)
Tracheostomy dependent	5	(22.7%)
VHI-10 (n = 17)	18.41 ± 11.30	
FOSS (n = 21)	0.33 ± 0.66	

Stage by AJCC 8^th^ edition.

### 2.1 TORS Procedures

**Figure 2:**
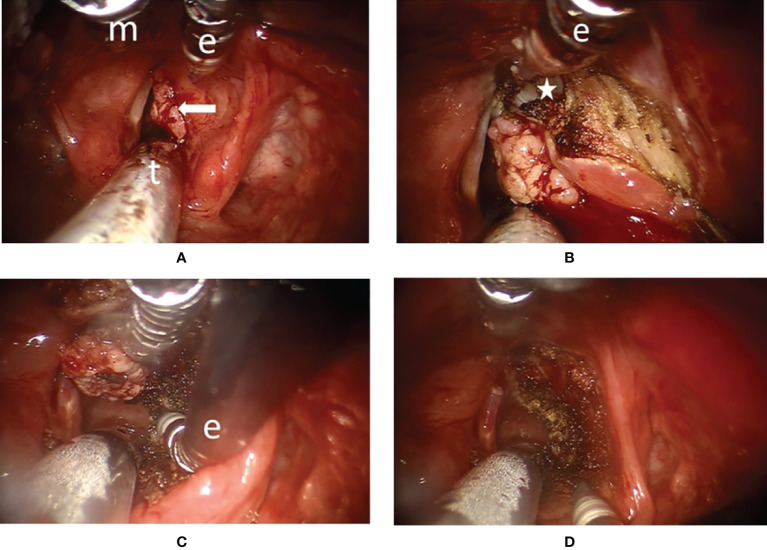
Surgical endoscopic view of transoral robotic surgery (TORS) for a type Va+c cordectomy in patient #13 **(A)** The panoramic view of a right vocal fold cancer with anterior commissure involvement (arrow). General anesthesia was delivered *via* the oral endotracheal tube (t). Maryland dissector (m) and monopolar electrocautery (e) were used to perform the dissection. **(B)** The spatula tip of the monopolar electrocautery (e) could be used to peel the perichondrium from the thyroid cartilage inner lamina (star) and to peel the Broyles’ ligament from the anterior commissure during superior part dissection. **(C)** The tip of the monopolar electrocautery (e) was angled anteriorly to cut along the upper border of the cricoid cartilage indicating the benefit of endo-wristed function. **(D)** After en-bloc resection of the tumor, the wound was clean with minimal bleeding.

All operations were performed by the corresponding author (C-CW) by the assistance of a second author (W-J-L) who is the assistant surgeon. The TORS was done under general anesthesia with a number 6.5 or 7.0 endotracheal tube inserted transorally and fixed on the patient’s mouth angle contralateral to the major tumor side in 21 patients. Only an 83-year-old male adult received elective tracheostomy for the surgery. The TORS technique has been introduced in our previous preliminary report (17). A 3D high-magnification endoscope (12-mm diameter in Si; 10-mm diameter in Xi) was inserted through the oral cavity, and the 2-instrument arms with a Maryland dissector (5-mm diameter in Si; 8-mm in Xi) and monopolar electrocautery (5-mm diameter in Si; 8-mm in Xi) were located at the left side and right sides, respectively, to perform the tumor resection. A 0° endoscope and a face-up 30° endoscope were used interchangeably to obtain a better view if necessary. An example of TORS en-bloc dissection of vocal fold tumor is shown in **Figure 2** and **Supplementary Video 1**, and several pieces of frozen section samples were harvested from the margins of the surgical wound until clear surgical margins were ensured. After the operation, a nasogastric tube (NG) tube was inserted as needed. The endotracheal tube was removed promptly if there was no significant laryngeal edema or several hours later after observing the airway.

### 2.2 Follow-Up and Data Analysis

**Figure 3:**
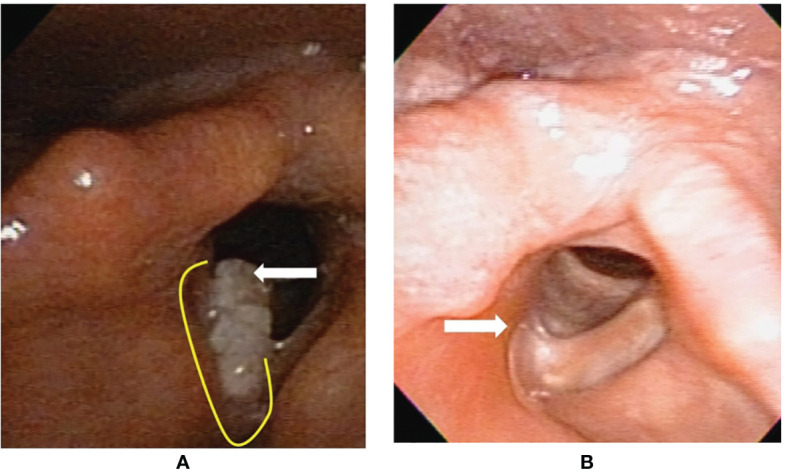
Flexible laryngoscopy view of patient #13 with fresh primary cancer before and after transoral robotic surgery (TORS) type Va+c cordectomy. **(A)** A cancer (arrow) involving the right vocal fold plus anterior commissure. The yellow line revealed the planned incision line surrounding the tumor. **(B)** After 25 months from TORS without adjuvant radiotherapy, a fibrotic scar (arrow) formed on the surgical wound bed without tumor recurrence.

**Figure 4:**
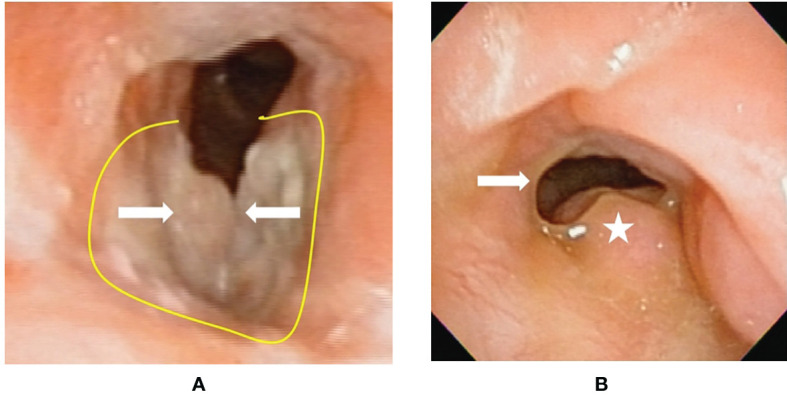
Flexible laryngoscopy view of patient #17 with recurrent cancer (after tran-soral laser microsurgery and radiotherapy failure) before and after transoral robotic surgery (TORS) type VI cordectomy. **(A)** A cancer (arrow) involving bilateral vocal folds plus anterior commissure. The yellow line revealed the planned incision line surrounding the tumor. **(B)** After 18 months from TORS without adjuvant radiotherapy, a fibrotic scar (arrow) formed on the right surgical wound bed. The left side false vocal fold had compensatory hypertrophy to maintain sphincter function at the glottis. There was no cancer recurrence.

After TORS, post-op adjuvant radiotherapy was not done because all final-cut margins of frozen specimens were negative for malignancy. Laryngeal wounds were observed closely by flexible laryngoscopy in the outpatient department until the wound healed (**Figures 3**, **4**). All the patients were followed up regularly to survey local recurrence, cervical regional recurrence, distant organ metastasis, or other new primary cancers. After long-term follow-up for at least 1 year for every case, survival analysis by the Kaplan–Meier method was used to determine the overall, disease-specific, and recurrence-free survival, etc. Overall survival was calculated from the date of TORS to the date of death or last follow-up. Disease-specific survival was calculated from the date of TORS to the date of disease-related death or last follow-up. Recurrence-free survival was measured from the date of TORS to the date of any evidence of recurrence or last follow-up. Factors that may be associated with survival were described and analyzed by t-test, chi-squared test, and Fisher’s exact test. The survival curves were compared for patients with primary cancer and recurrent cancer by the log-rank test.

In addition, for surviving patients, long-term functional evaluations such as larynx preservation rate, tracheostomy dependence rate, tube feeding rate, and phonation function recorded by Voice Handicap Index-10 (VHI-10) (19) and Functional Outcome Swallowing Scale (FOSS) (20) were also recorded. VHI-10 is a popular questionnaire to evaluate the function of voice with scores ranging from 0 to 40 (0–10: nearly normal voice; 11–20: mild dysphonia; 21–30: moderate dysphonia; 31–40 severe dysphonia). Similarly, FOSS is a popular questionnaire to evaluate swallowing with scores ranging from 0 to 5 (0: normal function and asymptomatic; 1: normal function but with episodic or daily symptoms of dysphagia; 2: compensated abnormal function manifested by significant dietary modifications or prolonged meal time; 3: decompensated abnormal function with weight loss of 10% or less; 4: decompensated abnormal function with weight loss of >10%; 5: non-oral feeding for all nutrition intake).

## 3 Results

There were totally 22 patients who received TORS in our study cohort. Twenty-one patients are male adults, and only one patient is female. Their ages ranged from 48 to 83 with mean ± SD of 66.55 ± 8.96. Eleven patients had fresh laryngeal cancers, and eleven patients had recurrent laryngeal cancers with history of endoscopic surgery (n = 4), radiotherapy (n = 5), or both (n = 2). Seven (31.82%) patients had clinical T1 tumors, and 15 (68.18%) patients had clinical T2 tumors. Only two patients had clinically suspected neck lymph node metastasis and received functional neck dissection. The rest of 20 patients received TORS only.

### 3.1 Perioperative Data

TORS was performed successfully in all 22 patients. Twenty patients received general anesthesia *via* an oro-endotracheal tube, and only an 83-year-old male received general anesthesia *via* tracheostomy due to old age with poor pulmonary function. Nineteen (86.36%) patients received type Va+c cordectomy, and 3 (13.64%) patients received type VI cordectomy (as classified by the European Laryngological Society) (21, 22). The pathologic T-stage equaled pathologic overall stages because there was no lymph node metastasis from the 2 patients receiving neck dissection. Nine (40.91%) patients had T1 disease, 11 (50%) patients had T2 disease, and 2 (9.09%) had T3 disease due to the inner cortex of thyroid cartilage which was involved in specimen. Ten (45.45%) patients had moderate cancer differentiation, 8 (36.36%) patients had moderate to poor cancer differentiation, and 4 patients had poor cancer differentiation. Thirteen (59.09%) had a negative margin on permanent specimen, and 9 (40.91%) patients had positive margins or undetermined margins due to torn permanent specimen. However, all patients had no malignancy from the margins of the frozen section at wound beds as mentioned in *Methods*. Two (9.09%) patients had lymphovascular invasion in the specimen. The data are summarized in **Table 1** for overall 22 patients, and in **Table 2** for different subgroups of patients with fresh cancer (n = 11), recurrent cancer with past history of RT (n = 7), and recurrent cancer without past history of RT (n = 4).

**Table 2:** The perioperative data and outcome differences between fresh cancer group (n = 11) and recurrent cancer group (n = 11).

	Fresh (n = 11)		Recurrence (n = 11)				p value
			Past RT (n = 7)		Without past RT (n = 4)		
	n	%	n	%	n	%	
Age (mean ± SD)	63.91	± 8.48	66.43	± 9.32	74.00	± 6.98	0.184
Sex							0.095
Female	0	(0%)	0	(0%)	1	(25%)	
Male	11	(100%)	7	(100%)	3	(75%)	
Clinical T stage							0.241
I	5	(45.45%)	2	(28.57%)	0	(0%)	
II	6	(54.55%)	5	(71.43%)	4	(100%)	
Clinical N stage*^c^*							0.225
0	10	(90.91%)	7	(100%)	3	(75%)	
I	1	(9.09%)	0	(0%)	0	(0%)	
IIc	0	(0%)	0	(0%)	1	(25%)	
Clinical stage*^c^*							0.228
I	5	(45.45%)	2	(28.57%)	0	(0%)	
II	5	(45.45%)	5	(71.43%)	3	(75%)	
III	1	(9.09%)	0	(0%)	0	(0%)	
IV	0	(0%)	0	(0%)	1	(25%)	
Cordectomy type							0.341
Va+_C_	10	(90.91%)	5	(71.43%)	4	(100%)	
VI	1	(9.09%)	2	(28.57%)	0	(0%)	
Pathologic stage*^c^*							0.058
I (T1N0)	6	(54.55%)	3	(42.86%)	0	(0%)	
II (T2N0)	5	(45.45%)	2	(28.57%)	4	(100%)	
III (T3N0)	0	(0%)	2	(28.57%)	0	(0%)	
Permanent specimen margin							0.075
Negative	9	(81.82%)	2	(28.57%)	2	(50%)	
Positive or undertermined	2	(18.18%)	5	(71.43%)	2	(50%)	
Lymphovascular invasion							0.279
No	11	(100%)	6	(85.71%)	3	(75%)	
Yes	0	(0%)	1	(14.29%)	1	(25%)	
Outcome (5 year)							
Death	0	(0%)	0	(0%)	1	(25%)	0.095
Disease-specific death	0	(0%)	0	(0%)	1	(25%)	0.095
Recurrence	0	(0%)	4	(57.14%)	1	(25%)	0.019*
Total laryngectomy	0	(0%)	3	(42.86%)	0	(0%)	0.024*
Tracheostomy dependent	0	(0%)	3	(42.86%)	2	(50%)	0.038*
VHI-10 (n = 17)	17.91	± 11.86	22.25	± 12.92	13.50	± 4.95	
FOSS (n = 21)	0.00	± 0.00	0.57	± 0.98	1.00	± 0.00	

Seven patients of the recurrent cancer group had past history of radiation failure.

^c^Chi-square test.

Fisher’s exact test.

*p < 0.05.

Except the 83-year-old patient with tracheostomy, 16 patients were extubated promptly after the TORS, and the endotracheal tubes of the rest of the 5 patients were re-moved after a 2-day observation. There were no devastating complications after surgery such as bleeding or airway emergence. Only a patient with chronic renal failure under regular hemodialysis had mild pneumonia, but it improved after antibiotic treatment. The hospitalization days ranged from 2 to 24 days with mean ± SD of 9.36 ± 7.58 days. Nasogastric (NG) tubes were inserted for feeding in 18 (81.8%) patients after surgery. Except the aforementioned 83-year-old male with recurrent cancers from past endoscopic surgery and another 56-year-old male with recurrent cancers from past radiotherapy which had prolonged NG placement for 5 and 7 months, respectively, the other 16 patients had NG for mean ± SD of 9.36 ± 7.58 days.

### 3.2 Oncologic Results

During TORS, frozen sections from 2 to 8 pieces with mean ± SD of 4.8 ± 1.4 pieces were sent for confirming the cut margins are clear from malignancy. After TORS, all patients were followed up regularly at our outpatients’ department. The follow-up duration ranged from 17 to 124 months with mean ± SD of 54.7 ± 37.2 months. The medium follow-up was 40.1 months.

**Figure 5:**
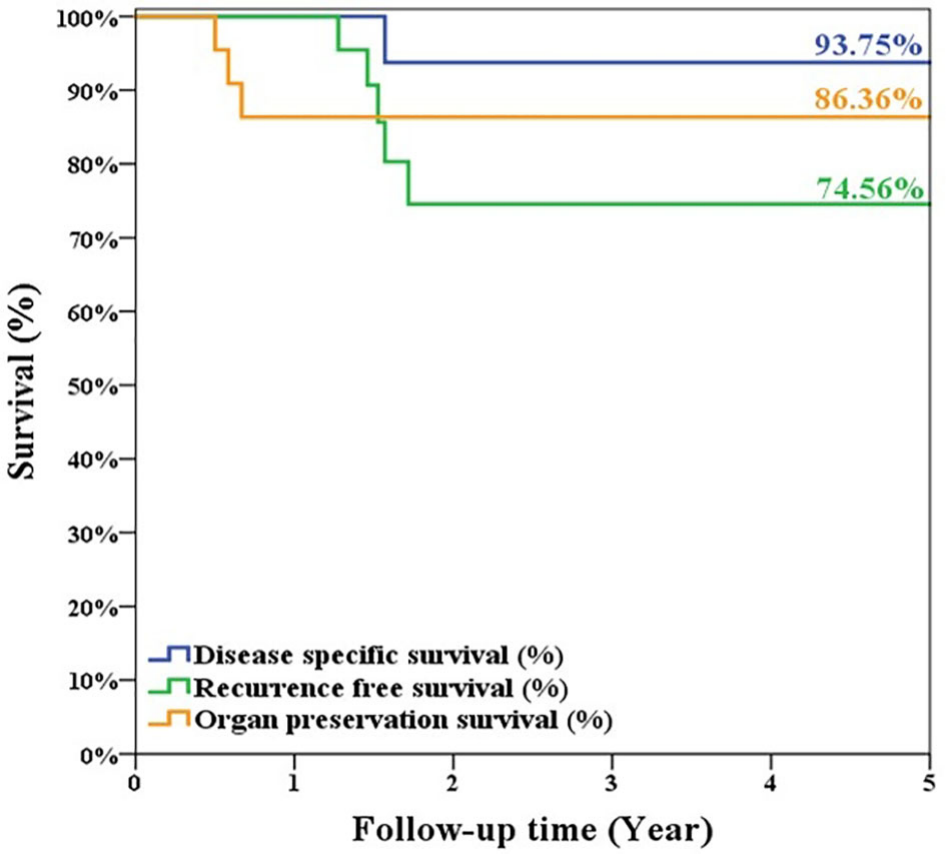
The estimated 5-year overall survival/disease-specific survival rate was 93.75%, the recurrence-free survival rate was 74.56%, and organ preservation rate was 86.36% for the 22 patients’ cohort of glottic cancer with anterior commissure involvement after TORS.

**Figure 6:**
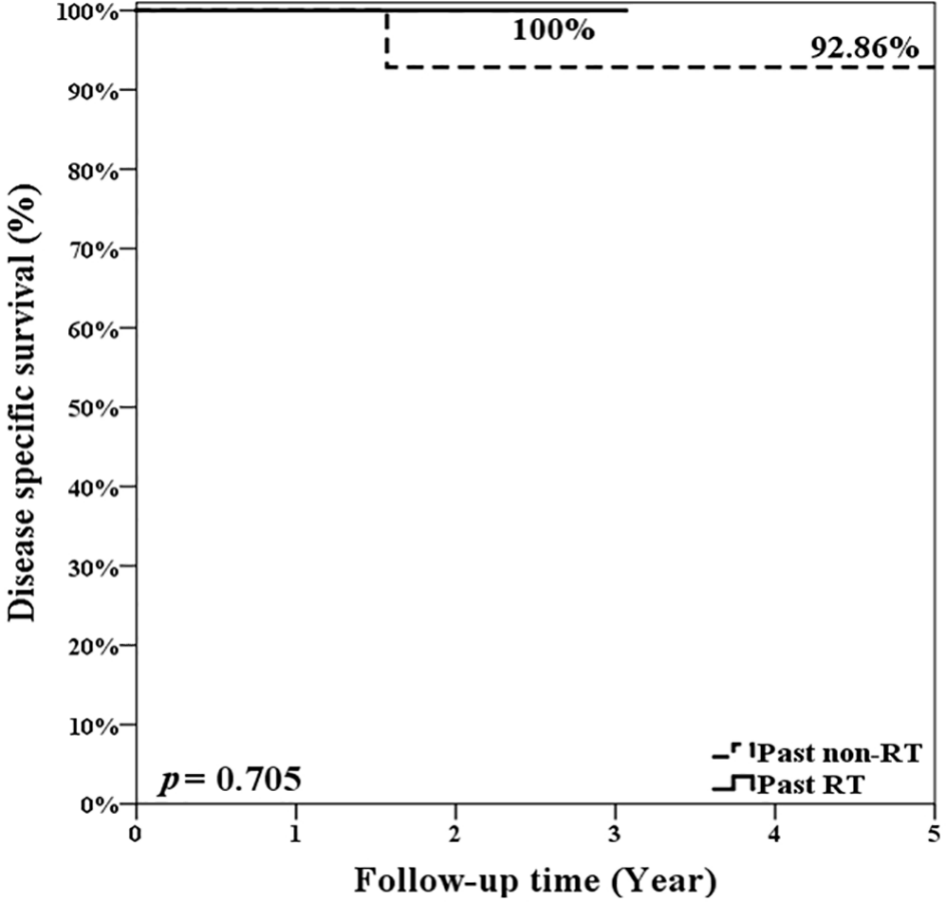
The estimated 5-year overall survival rate and disease-specific survival rate were 92.86% for patients without history of radiotherapy and were 100% for patients with history of radiotherapy. There was no significant difference (p = 0.705; log rank test).

During follow-up, 5 patients experienced primary recurrence. The recurrence time ranged from 4 to 17 months after surgery with mean ± SD of 8.8 ± 5.1 months. Three patients received TL for salvage and are alive without disease now. The other 2 patients refused total laryngectomy. A patient received radiotherapy but died from lung metastasis 19 months after TORS. Another alive patient with history of radiation failure refused any treatment and accepted symptom treatment only. The 5-year overall survival rate of 93.8% (equaled disease-specific survival curve), recurrence-free survival rate of 74.6%, and organ preservation rate of 86.4% were estimated by the Kaplan–Meier method and are shown in **Figure 5**. The oncologic outcomes were quite satisfactory. The data comparing the outcomes of TORS for patients with/without history of RT are summarized in **Table 3**, and the 5-year estimated overall survival curve and disease-specific survival curve higher than 90% for both groups are displayed in **Figure 6**. However, the recurrence and salvage total laryngectomy rate were much higher in patients with history of radiotherapy (**Table 3**).

**Table 3:** The outcomes of 22 patients and the differences between patients with and without previous history of radiotherapy.

Outcome (5 year)	No previous RT (n = 15)		Previous RT (n = 7)		p value
	n	%	n	%	
Death	1	(6.67%)	0	(0%)	1.000
Disease-specific death	1	(6.67%)	0	(0%)	1.000
Recurrence	1	(6.67%)	4	(57.14%)	0.021*
Total laryngectomy	0	(0%)	3	(42.86%)	0.023*
Tracheostomy	2	(13.33%)	3	(42.86%)	0.274
VHI-10 (n = 17)	17.23	± 11.05	22.25	± 12.92	
FOSS (n = 21)	0.21	± 0.43	0.57	± 0.98	

Fisher’s exact test.

*p < 0.05.

### 3.3 Functional Outcomes

After long-term follow-up, 3 patients had permanent tracheostoma due to total laryngectomy, and 1 patient had tracheostomic tube before his death. Another patient had laryngeal stenosis after the surgery and had continuous tracheostomic tube insertion. The VHI-10 of 17 patients without tracheostomy ranged from 1 to 37 with mean ± SD of 18.41 ± 11.30. The results indicated that most of the patients had mild dysphonia after the TORS. Except the expired patient, the rest of the 21 patients including 3 total laryngectomees answered the questionnaire of FOSS. Sixteen patients had a score of 0, 3 patients had a score of 1, and 2 patients had a score of 2. There were no patients with scores of 3, 4, and 5. The mean ± SD of FOSS is 0.33 ± 0.66. The results indicate that all the patients could have normal, nearly-normal, or compensated swallowing function.

## 4 Discussion

The impact of the AC involvement on prognosis in early T1 and T2 laryngeal cancer remains a topic of debate. A recent review found that the past studies were too heterogeneous to perform a formal meta-analysis and no definite conclusion could be drawn from their review. However, the weighted averages from the study showed that the involvement of AC leads to a slightly higher recurrence rate after treatment with both RT and TLM (23). The systematic review and meta-analysis of Tulli et al. (24) pointed out that AC involvement in T1 glottic cancer had a significant lower 5-year local control rate and specific discussion on the treatment results of early glottic cancer with AC involvement should be recommended.

### 4.1 RT, TLM, and PL for Glottic ca With AC Involvement

As mentioned in *Introduction*, PL is mostly reserved for laryngeal recurrence at the present time (6, 7). RT and TLM are both popular initial treatments for early laryngeal cancer with AC involvement, but TLM had achieved a higher rate of overall survival laryngeal preservation in a systematic review and meta-analysis (25). Although Steiner et al. (8) reported good results of TLM, Wolfensberger et al. believe that laser surgery should be avoided in case of AC involvement because of a high rate of recurrence (26). Peretti et al. (27) pointed out that adequate exposure of the glottic larynx is sometimes a serious limitation in TLM. Desloge et al. (28) have also emphasized the need for wide exposure when using endoscopic techniques to safely narrow cancer-free margins. Our TORS procedure therefore could be considered as a salvage transoral approach if adequate exposure could not be achieved in TLM.

### 4.2 TORS for Glottic ca in Literature

The feasibility of TORS in glottic surgery was first demonstrated by O’Malley et al. in a canine model in 2006 (11). In 2009, Park et al. (12) described the feasibility of TORS on 2 patients with glottic ca involving the AC without reporting the long-term results. From 2009 to 2019, 7 papers described TORS for glottic cancers and 5 of the 7 papers have mentioned the results for AC involvement from 1 to 8 cases. The study designs including temporary tracheostomy and long-term follow-up duration are summarized in **Table 4**. In 2016, we published the first paper focused on using TORS without tracheostomy for 8 patients of early glottic ca with AC involvement (17). Without post-TORS adjuvant radiotherapy, there was no local recurrence after a mean follow-up of 40 months. The functions after TORS were satisfactory. The present study could offer the update of bigger cohort and news of TORS for AC involvement either in fresh cases or in recurrent cases even after irradiation failure.

**Table 4:** TORS for glottic ca with or without AC involvement in literature.

Year	Author (reference)	Case no.	AC case no.	Tracheostomy	Long-term outcomes
2009	Park (12)	4	2	Yes	Not available
2011	Blanco (13)	1	1	Yes	Not available
2012	Vural (18)	1	1	Yes	6-months
2012	Kayhan (14)	10	Not described	Done in 1 patient	Ranged from 2 to 16 months
2013	Lallemant (16)	13	Not described	Done in 1 patient	12 months
2016	Wang (17)	8	8	Nil	Mean follow-up 40 months
2019	Kayhant (15)	48	6	Nil	Mean follow-up 65 months
2021	Present study	22	22	Done in 1 patient	Mean follow-up 55 months

### 4.3 Technical/Oncological Safety and Organ/Function Preservation of TORS

From our results, the TORS could be safe without major complications and almost all the procedures could be performed without temporary tracheostomy like TLM. The 5-year local control and disease-free survival rate were both 100% in our 11 patients with fresh glottic cancer. The results looks better than the reports of TLM by Rodel et al. (3) and Hoffmann et al. (29), but our cohort was much smaller than theirs. Their data and ours are summarized in **Table 5**. For overall 22 patients including recurrence cases, the recurrence-free survival was 74.56% which was similar to the data of TLM for fresh cases. Our recurrence-free survival was even higher (92.86%) in patients without previous history of radiation failure. In 2017, Stephenson et al. (30) reported that they used TLM with 40% adjuvant radiotherapy to get 96.7% organ preservation for 30 patients with glottic ca and AC involvement. In their review of other studies, the laryngeal preservation rate ranged from 88% to 98.8% in different studies with various study designs. Our organ preservation rate was 100% for patients without history of irradiation failure and was 86.36% for both untreated and recurrent patients.

**Table 5:** Results of transoral surgery for fresh case of glottic cancer with AC involvement.

Year	Author (reference)	Approach	Chohor	Results
2009	Rodel (3)	TLM	n = 153	5-year local control
			T1	73% for T1a,
			T2	68% for T1b,
				76% for T2
2016	Hoffman (29)	TLM	n = 96	5-year local control 74.4%
			Tis	5-year disease free survival 61.7%
			T1	5-year overall survival 79.2%
			T2	5-year disease specific survival 91.5%
2021	Present study	TORS	n = 11	5-year local control 100%
			T1	5-year disease free survival 100%
			T2	5-year overall survival 93.8%
				5-year disease specific survival 93.8%

### 4.4 TLM for Post Irradiation Recurrent Glottic Cancer With AC Involvement

As mentioned in *Introduction*, AC involvement is considered a predictor of poor response to RT (1, 31). In 2004, Puxeddu et al. (10) used TLM for salvage surgery after radiotherapy failure in 16 patients of T1 and T2 glottic carcinoma. Thirteen (81.25%) patients had successful salvage TLM. The 3-year local control with laser alone was 87.1%. Only 4 (25%) patients had type V cordectomy encompassing AC in their cohort. For their 4 patients after type V cordectomy, 2 (50%) patients had local recurrence that needed further treatment but only one (25%) patient died of disease on follow-up. In the same year, Steiner et al. (32) reported their results and reviewed similar studies on TLM for recurrent glottic carcinoma after radiotherapy. In their 21 patients of AC involvement after radiotherapy, 12 (57%) patients had a second recurrence and 6 (28.6%) of them received following total laryngectomy. Steiner et al. concluded that the number of salvage laryngectomies in patients with recurrences extending into the AC was higher than in patients without commissural disease. Cancers that recur after irradiation failure often demonstrate aggressive behavior, arise in a field where lymphatic drainage is unpredictable, and are associated with poor control rate (33, 34). That is why in other studies (35–40), the salvage laryngectomy rate ranged from 13% to 53% and the cure rate also varied a lot although the study subjects were not all having recurrent cancer with AC involvement. However, Steiner et al. (32) found that the favorable functional results and rare postoperative complications after TLM contribute to less hospitalization than with conventional partial and total laryngectomy. Therefore, TORS may also be an alternative of transoral surgery after irradiation failure for glottic cancer if the TORS could offer better exposure than TLM (**Figure 1**).

### 4.5 TORS for Post Irradiation Recurrent Glottic Cancer With AC Involvement

In the previous discussion, we already demonstrated the oncologic safety of using TORS on recurrent glottic cancer with AC involvement and the 5-year disease-specific survival is 93.75%. When we focused on our 7 patients after irradiation failure, 4 (57.1%) patients had local recurrence after TORS and then 3 (42.9%) patients had salvage laryngectomy, but all patients survived. However, compared to patients without history of radiotherapy, the group after irradiation failure had a significant higher recurrence rate and lower organ preservation but the overall and disease-specific survival is similar (**Table 3** and **Figure 6**). It seems that, before salvage open PL or TL, TORS is also a feasible minimally invasive surgery for glottic cancer with AC involvement after recurrence from TLM, or even from radiotherapy, without compromising future survival. However, the rate of second recurrence could be higher in salvage TORS and we need to mention the possibility of further open surgery to patients before performing TORS.

### 4.6 PL for Post Irradiation Recurrent Glottic Cancer With AC Involvement

While TLM and TORS had lower local control rates for recurrence at AC after irradiation failure, probably open PL which encompasses thyroid cartilage excision may provide better local control. Recently, De Virgilio et al. (41) did a meta-analysis and reported an 85.2% laryngeal preservation rate of radio-recurrent laryngeal cancer treated by supracricoid PL. In a multicenter study reported by Bertolin et al. (42), the local control rate was 87% and the overall survival was 75%. However, the authors concluded that PL should not be considered as the standard for salvage of recurrence after radiotherapy failure. In the choice between PL and TL for salvage, not only tumor-related factors (clinical stage) but also the patient’s general conditions must be considered (42). Holsinger et al. (43) also indicated that the patient must present good pulmonary function, no major comorbidities, and willingness to accept potential lengthy rehabilitation if PL was going to be done. Because 17% of the neo-laryngeal stenosis rate, 10% of wound infection rate, 7% of pharyngocutaneous fistula rate, and 7% of dysphagia rate were not neglectable in the multicenter study (42). Succo et al. (44) pointed out the limitations of open PL in the fact that postoperative management is often difficult and residual laryngeal function can vary greatly among centers (45). Particularly, the quality of vocal recovery can vary immensely, even in the hands of experienced surgeons (44).

### 4.7 Study Limitations

This study had several limitations.

#### 4.7.1 Small Cohort Size

First, the cohort size was small (n = 22). However, only 20% of glottic cancer showed AC involvement, and not every patient chose transoral surgery as initial treatment. In addition, TORS is a new treatment modality. Therefore, it is difficult for a single institute to recruit a large number of patients for study. Further large-scale or multi-institutional studies are necessary to confirm this news and updated results. Our study is so far the paper with the largest cohort reporting on TORS for glottic cancer involving AC.

#### 4.7.2 Optimal Exposure Not Always Available

Second, good exposure for TORS is not always possible especially for patients with small mouth, long teeth, big tongue, retrognathia, or c-spine problems. However, those patients are not good candidates for TLM either. If the exposure was better in TORS in the trial test we have proposed (**Figure 1**), TORS should be a better option for surgery. Kayhan et al. (14) also commented that en-bloc excision is difficult along the narrow laryngoscope space in TLM. However, en-bloc excision could be achieved with safe surgical margins if TORS exposure had been assured in the trial test.

#### 4.7.3 Instrument Limitation

Third, the size of endo-wristed instruments of da-Vinci robot is still not optimal for transoral surgery and we expect that TORS will be more efficient in glottic surgery if the da-Vinci system would downsize the instruments. However, it relies on us to report the feasibility of TORS in this field, and therefore we could urge the company to develop more specific instruments for ENT doctors in the future. Some surgeons may concern about the thermal injury caused by electrocautery; however, we could minimize the injury by using the spatula tip of electrocautery to peel the perichondrium and Broyles’ ligament off thyroid cartilage which is clearly shown in **Figure 1B** and the supplement video. Probably that is the reason why Kahan et al. claimed that they did not encounter thermal injury in their series. Furthermore, thermal injury could also be reduced by adding laser technology to the robotic arm (15). In our opinion, there is no big difference in thermal injury between electrocautery and CO_2_ laser when we have to make a deep resection that reaches the thyroid cartilage at the anterior commissure (17).

#### 4.7.4 Consideration of Airway and Swallowing

Fourth, 5 (22%) of our patients had intubation for up to 48 h and nasogastric tubes were placed in all patients with an average duration of 9 days. It seems to be a disadvantage of TORS. In the study of TLM for recurrent glottic cancer reported by Puxeddu et al. (10), no feeding tube was inserted and their mean hospitalization time was 2.2 days. However, 12 (75%) of their 16 patients had T1 cancer and 4 (25%) patients had T2 cancer. Conversely, 32% of our patients had T1 cancer and 68% of our patients had T2 cancer. A bigger tumor may lead to larger wounds and more morbidities including laryngeal edema and odynophagia. However, longer duration of intubation in some patients and temporary nasogastric tube insertion did not lead to worse function in our study in the long-term follow-up. In the report of TORS by Lallemant et al. (16), a patient had pT2 cancer extending to the supraglottic region who experienced cervical emphysema, pneumothorax, and laryngeal bleeding 5 h after TORS. The patient received tracheostomy, bleeding check-up endoscopically, and chest tube insertion. Therefore, for larger type V or VI cordectomy, individual consideration on airway and feeding should be made according to different condition after TORS.

#### 4.7.5 Cost of Robotic Surgery

Finally, the cost burdens are still a major disadvantage of TORS. However, while we evaluate cost-effectiveness on different surgical approaches, the long-term economic aspects of advantages are not considered. Dombree et al. (40) had suggested that further research is needed to gather enough data for evaluating long-term costing. In the paper of Kayhan et al. (14), they commented that using TORS in other branches of medicine increases, the cost will decrease. Hopefully, this paper had contributed some on improving TORS and that more economical robotic systems may come through marketing developments and corporation competition. Consequently, the cost should be reduced in the future.

## 5 Conclusions

TORS could be eligible in the primary or salvage management of glottic cancer with AC involvement while TORS was confirmed to have better exposure to TLM. The 5-year overall survival rate of 93.8% (equaled disease specific survival curve) was satisfactory. In the subgroup of patients having recurrent cancer after irradiation failure, TORS could also be a minimally invasive alternative before trying open surgery to preserve the organ and reduce the morbidity. Their swallowing function was good, and voice function was serviceable for daily life with mild impairment. If unfortunately, tumor recurred again, the patient could be rescued by TL without compromising the estimated 5-year survival.

## Data Availability Statement

The raw data supporting the conclusions of this article will be made available by the authors, without undue reservation.

## Ethics Statement

The studies involving human participants were reviewed and approved by the Institutional Review Board of Taichung Veterans General Hospital (IRB TCVGH No. CE20389B). Written informed consent for participation was not required for this study in accordance with the national legislation and the institutional requirements.

## Author Contributions

W-JL worked together with C-CW to perform surgeries on all 22 patients with acquisition, analysis, interpretation of data, and drafting of the manuscript. C-CC helped in performing radiotherapy when patients had tumor recurrence and in conceptualization of the paper. J-JW, K-LL, and Y-JH helped with the conceptualization of the paper, with the referral of patients, and with administrative, technical, or material support, and gave recommendations on the writing of the manuscript. All authors contributed to the article and approved the submitted version.

## Conflict of Interest

The authors declare that the research was conducted in the absence of any commercial or financial relationships that could be construed as a potential conflict of interest.

## Publisher’s Note

All claims expressed in this article are solely those of the authors and do not necessarily represent those of their affiliated organizations, or those of the publisher, the editors and the reviewers. Any product that may be evaluated in this article, or claim that may be made by its manufacturer, is not guaranteed or endorsed by the publisher.

## Supplementary Material

Supplementary Video 1 Transoral robotic surgery for a T1 glottic cancer with anterior commissure involvement. The video shows the procedure of using Transoral Robotic Surgery (TORS) *via* da-Vinci robot Si-system to resect a T1 glottic cancer located at the right-side vocal fold with anterior commissure involvement. Under general anesthesia with oro-endotracheal intubation, glottis was exposed by Laryngeal Advanced Retractor System (LARS). With a panoramic view, the tumor could be en-bloc resected by using endo-wristed instruments (Maryland dissector at the left-hand side and Mono-polar cautery at the right-hand side). The spatula tip of mono-polar cautery could be used to peel the Broyle’s ligament from the inner lamina of thyroid cartilage and the bleeding during dissection could be easily stopped by using electrocauterization. The video has been published in Zenodo.org DOI:10.5281/zenodo.4570201. https://zenodo.org/record/4570201#.YQ-cRogzZEA.
